# Symbiosis of Electrical and Metabolic Oscillations in Pancreatic β-Cells

**DOI:** 10.3389/fphys.2021.781581

**Published:** 2021-12-03

**Authors:** Isabella Marinelli, Patrick A. Fletcher, Arthur S. Sherman, Leslie S. Satin, Richard Bertram

**Affiliations:** ^1^Centre for Systems Modelling and Quantitative Biomedicine (SMQB), University of Birmingham, Birmingham, United Kingdom; ^2^Laboratory of Biological Modeling, National Institutes of Health, Bethesda, MD, United States; ^3^Department of Pharmacology, Brehm Center for Diabetes Research, University of Michigan Medical School, Ann Arbor, MI, United States; ^4^Programs in Neuroscience and Molecular Biophysics, Department of Mathematics, Florida State University, Tallahassee, FL, United States

**Keywords:** metabolism, oscillations, calcium, bursting, insulin

## Abstract

Insulin is secreted in a pulsatile pattern, with important physiological ramifications. In pancreatic β-cells, which are the cells that synthesize insulin, insulin exocytosis is elicited by pulses of elevated intracellular Ca^2+^ initiated by bursts of electrical activity. In parallel with these electrical and Ca^2+^ oscillations are oscillations in metabolism, and the periods of all of these oscillatory processes are similar. A key question that remains unresolved is whether the electrical oscillations are responsible for the metabolic oscillations via the effects of Ca^2+^, or whether the metabolic oscillations are responsible for the electrical oscillations due to the effects of ATP on ATP-sensitive ion channels? Mathematical modeling is a useful tool for addressing this and related questions as modeling can aid in the design of well-focused experiments that can test the predictions of particular models and subsequently be used to improve the models in an iterative fashion. In this article, we discuss a recent mathematical model, the Integrated Oscillator Model (IOM), that was the product of many years of development. We use the model to demonstrate that the relationship between calcium and metabolism in beta cells is symbiotic: in some contexts, the electrical oscillations drive the metabolic oscillations, while in other contexts it is the opposite. We provide new insights regarding these results and illustrate that what might at first appear to be contradictory data are actually compatible when viewed holistically with the IOM.

## Introduction

Pancreatic β-cells contribute to maintaining glucose homeostasis in the body by producing and secreting insulin in a pulsatile fashion, with a period of 4–5 min (Laurenti et al., 2020, 2021; Shukla et al., 2021). Pulses of insulin are more efficient than constant secretion at lowering the level of plasma glucose by suppressing hepatic glucose production and stimulating uptake of glucose by muscle and adipose tissue (Komjati et al., 1986; Paolisso et al., 1991; Matveyenko et al., 2012), and plasma insulin oscillations are disrupted in patients with type I and II diabetes (reviewed in Satin et al., 2015). Insulin secretion is evoked by elevations in the intracellular Ca^2+^ concentration, so pulsatile insulin secretion is associated with oscillations in the intracellular Ca^2+^ concentration.

This has been studied most directly in mice, where it has been possible to measure both *in vivo* insulin oscillations and *in vitro* calcium oscillations from the same mouse and show that the frequencies are well correlated (Nunemaker et al., 2005). Although the oscillation patterns are more varied and complex in humans, slow oscillations of appropriate frequency in calcium (Martin and Soria, 1996; Bertram et al., 2018), membrane potential (Riz et al., 2014), and insulin secretion (Marchetti et al., 1994; Lin et al., 2002; Misun et al., 2020) have been observed in human islets *in vitro*. Thus, these oscillations all originate from bursting electrical activity. In addition, they are accompanied by oscillations in metabolism, as demonstrated by multiple observations in mouse islets of ATP concentration (Li et al., 2013; Merrins et al., 2016), NAD(P)H fluorescence (Luciani et al., 2006; Merrins et al., 2010), mitochondrial membrane potential (Krippeit-Drews et al., 2000; Merrins et al., 2016), and the glycolytic metabolite fructose-1,6-bisphosphate (FBP) (Merrins et al., 2013, 2016).

Bursting electrical activity consists of an episode of action potentials (the *active phase*) followed by a period of membrane repolarization (the *silent phase*), repeated periodically. Since the electrical impulses are produced by Ca^2+^ influx through voltage-dependent Ca^2+^ channels (Ca^2+^ channels), the intracellular Ca^2+^ concentration is higher during each burst active phase and lower during each silent phase. These periodic rises in Ca^2+^ evoke exocytosis of insulin-containing vesicles during each active phase (Rorsman and Ashcroft, 2018).

The mechanism responsible for bursting activity remains elusive. It has been proposed that Ca^2+^ oscillations are responsible for periodically switching the cell between the active and silent phases by acting on Ca^2+^-dependent K^+^ channels [K(Ca) channels], or (indirectly) on ATP-sensitive K^+^ channels [K(ATP) channels] (Chay and Keizer, 1983; Keizer and Smolen, 1991; Smolen and Keizer, 1992; Magnus and Keizer, 1998; Fridlyand et al., 2005; Diederichs, 2006; Cha et al., 2011). However, alongside these Ca^2+^ oscillations are metabolic oscillations that have a similar period. These oscillations are manifested as oscillations in the ATP/ADP ratio, which determines K(ATP) channel activity (Ashcroft et al., 1984; Cook and Hales, 1984; Henquin, 1988), thus linking oscillations in metabolism to oscillations in electrical activity and Ca^2+^. Whether these metabolic oscillations are intrinsic (Tornheim, 1997) or passively driven by the downstream effects of Ca^2+^ oscillations (Fridlyand et al., 2005; Cha et al., 2011) has been a matter of debate. One proposal is that metabolic oscillations are driven by oscillations in glycolysis (Tornheim, 1997), as has been demonstrated in extracts from skeletal muscle cytosol (Tornheim and Lowenstein, 1974). Another is that ATP/ADP varies dynamically due to the hydrolysis of ATP to power the Ca^2+^ pumps of the plasma and ER membranes. These pumps remove Ca^2+^ from the cytosol during each active phase (Detimary et al., 1998).

Although both the cytosolic Ca^2+^ concentration and ATP (and other metabolites) oscillate with similar periods, the question of “who is driving bursting?” has yet to be answered. Are Ca^2+^ oscillations driving metabolic oscillations, or vice versa? Mathematical modeling provides a valuable tool for addressing this question, as we demonstrate herein. Models can incorporate different oscillation mechanisms that yield differential predictions. One model, the Integrated Oscillator Model (IOM) (McKenna et al., 2016; Marinelli et al., 2018), incorporates both bursting mechanisms, with different parameter combinations determining which mechanism wins out. In this perspectives article, we use the IOM to demonstrate how the model has been and can be used to investigate the origin of pulsatile insulin secretion, electrical bursting oscillations, and Ca^2+^ and metabolic oscillations of pancreatic β-cells. We also use the IOM to demonstrate how modeling can resolve apparently contradictory data concerning the effects of Ca^2+^ on the polarization of the mitochondrial inner membrane.

## Materials and Methods

### Mathematical Model

**FIGURE 1:**
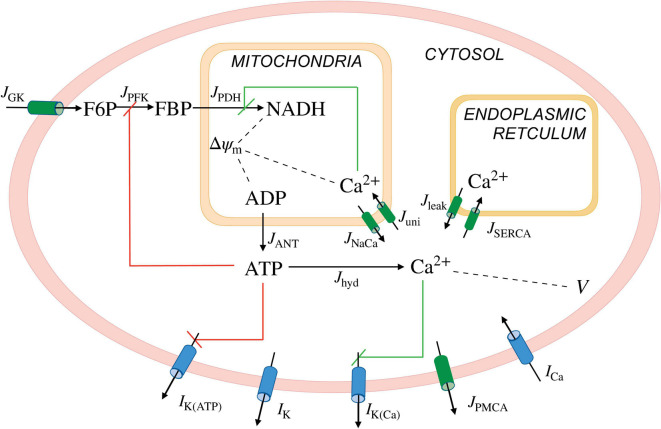
Illustration of the *IntegratedOscilla*tor*Model*(*IOM*). The model consists of three modules. A first module describes the β-cell electrical activity and Ca^2+^ dynamics, the second describes key elements of glycolysis, and the third accounts for the mitochondrial metabolism. The second and third modules together make up the metabolic subsystem. ***I***, ionic currents; ***J***, fluxes; K(ATP), ATP-sensitive K^+^ channels; K, delayed rectifier K^+^ channels; K(Ca), Ca^2+^-activated K^+^ channels; PMCA, plasma membrane Ca^2+^ ATPase; Ca, *V*-dependent Ca^2+^ current; GK, glucokinase; PFK, phosphofructokinase; PDH, phosphate dehydrogenase; ANT, adenine nucleotide translocator; hyd, hydrolysis; NaCa, Na^+^-Ca^2+^ exchanger; uni, uniporter; leak, Ca^2+^ leak across the ER membrane; SERCA, sarcoplasmic endoplasmic reticulum Ca^2+^ pumps; *V* and △ψ_*m*_, cellular and mitochondrial membrane potential, respectively. The red and green lines represent negative and positive feedback, respectively.

We used the Integrated Oscillator Model (IOM) to simulate a representative β-cell in a well-coupled islet. Figure 1 shows key elements of the model. The current model is an extension of a prior IOM implementation (Marinelli et al., 2018). In particular, we added a third module to account for mitochondrial metabolism (adapted from Bertram et al., 2007a) to the previous two modules representing cellular electrical activity and glycolysis. The differential equations were integrated numerically using MATLAB (MathWorks Inc., Natick, MA); the computer code can be downloaded from https://www.math.fsu.edu/∼bertram/software/islet/.

In this model, the flux through the pyruvate dehydrogenase (PDH) reaction (*J*_*PDH*_) is given by


(1) $$J_{\text{PDH}}=v_{\text{PDH}}\,\frac{1}{K_{{\text{NADH}}_{\text{m}},\mathrm{PDH}}+\frac{{\text{NADH}}_{\text{m}}}{{\text{NAD}}_{\text{m}}}}\,J_{\text{GPDH}},$$

where *v*_*PDH*_ is the maximum PDH reaction rate, *J*_*GPDH*_ is the flux through the glycerol-3-phosphate dehydrogenase (GPDH) reaction, and NADH_*m*_ and NAD_*m*_ are dinucleotides of mitochondria. The GPDH reaction rate (*J*_*GPDH*_) is given by


(2) $$J_{\text{GPDH}}=\frac{{\text{Ca}}_{\text{m}}^{2+}}{{\text{K}}_{\text{GPDH}}+{\text{Ca}}_{\text{m}}^{2+}}\,\sqrt{\text{FBP}},$$

with ${\text{Ca}}_{\text{m}}^{2+}$ representing the Ca^2+^ concentration of the mitochondria. In the IOM, the K(ATP) current (*I*_*K(ATP)*_) is described by


(3) $$I_{\text{K}\,\left(\text{ATP}\right)}=g_{\text{K}\,\left(\text{ATP}\right)}\,o_{\infty }\,\left(\mathrm{ATP},\mathrm{ADP}\right)\,\left(V-V_{\text{K}}\right),$$

where *g*_*K(ATP)*_ is the maximum conductance, *V* is the plasma membrane potential, *V*_*K*_ is the K^+^ Nernst potential, and *o*_∞_(*ATP*,*ADP*) is the fraction of activated K(ATP) channels,


(4) $$o_{\infty }\,\left(\mathrm{ADP},\mathrm{ATP}\right)=\frac{0.08+0.89\,{\left(\frac{\text{MgADP}}{k_{d\,d}}\right)}^{2}+0.16\,\left(\frac{\text{MgADP}}{k_{d\,d}}\right)}{{\left(1+\frac{\text{MgADP}}{k_{d\,d}}\right)}^{2}\,\left(1+\frac{{\text{ATP}}^{4-}}{k_{t\,t}}+\frac{{\text{ADP}}^{3-}}{k_{t\,d}}\right)}.$$

Here, MgADP = 0.165 ADP, ADP^3–^ = 0.135 ADP, and ATP^4–^ = 0.05 ATP, while the parameters *k*_*tt*_, *k*_*td*_ and *k*_*dd*_ represent the dissociation constants for ATP, ADP, and MgADP, respectively.

We simulated the addition of the K(ATP) channel activator diazoxide (Dz) by increasing the dissociation constants for ATP (*k*_*tt*_) from 1 to 2μM. The application of KCl, which induces cell depolarization, was simulated by increasing the K^+^ Nernst potential (*V*_K_) from –75 to −70*mV*.

The model used here should be thought of as simulating a representative β-cell in a well synchronized islet. There has been a great deal of interest recently in β-cell heterogeneity within the islets, which is beyond our scope, as it doesn’t generate the oscillations, though it does strongly influence the characteristics of the oscillations. The hypothesis that the mechanisms of oscillations are inherent in the β-cells rather than the network is supported by calcium imaging in isolated β-cells and in islets lacking gap junctions. Oscillations are observed but are irregular (likely due to noise) and occur out of the normal glucose range (likely due to heterogeneity) and, in islets, asynchronous (Ravier et al., 2005; Benninger et al., 2011; Dwulet et al., 2019; Scarl et al., 2019). See the following for recent work on the roles of heterogeneity (Cappon and Pedersen, 2016; Benninger and Hodson, 2018; Nasteska and Hodson, 2018; Stozer et al., 2019; Joglekar et al., 2021; Zmazek et al., 2021).

## Results

### Electrical, Ca^2+^, and Metabolic Subsystems Are Symbiotic

**FIGURE 2:**
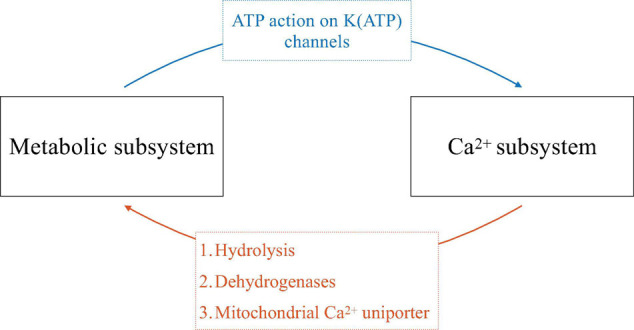
Schematic of the symbiosis of electrical and metabolic oscillators. The ways that one subsystem affects the other is indicated on the arrows.

The transduction of glucose input to insulin output in β-cells is complex, and can be thought of in terms of feedforward and feedback pathways implemented through the metabolism of glucose (which, unlike most cells, plays a key role not only in providing energy but also in intracellular signaling), intracellular Ca^2+^ signaling, and membrane electrical activity. We describe these pathways in some detail below, but begin with an overview illustrated in Figure 2.

Glucose triggers β-cell electrical activity through the metabolic production of ATP from ADP, resulting in an increase in cytosolic ATP/ADP. This increase inhibits K(ATP) channels and thereby reduces net outward K^+^ current, resulting in cell depolarization. This depolarization opens voltage-dependent Ca^2+^ channels and allows Ca^2+^ influx, producing electrical impulses and increasing the intracellular Ca^2+^ concentration (blue arrow, Figure 2). In this way, metabolism feeds forward onto the cell’s electrical activity and Ca^2+^ subsystem. If metabolism oscillates, this pathway can produce bursting electrical activity and Ca^2+^ oscillations.

Cytosolic Ca^2+^, as well as Ca^2+^ entering organelles such as the endoplasmic reticulum (ER) and mitochondria, in turn affects metabolism through several pathways (orange arrow, Figure 2). One is via ATP hydrolysis needed to power the Ca^2+^ pumps of the plasma membrane and ER membrane. These pumps, or Ca^2+^-ATPases, are particularly active during the active phases of bursting, when cytosolic Ca^2+^ is elevated. The hydrolysis of ATP that results in turn reduces cytosolic ATP/ADP. If the Ca^2+^ concentration oscillates, then ATP/ADP, and the levels of other metabolites, will concomitantly oscillate as ATP is hydrolyzed by Ca^2+^ pump activity (Detimary et al., 1998). A second way for Ca^2+^ to affect metabolism is by activating dehydrogenases of the glycolytic pathway and tricarboxylic acid cycle (TCA cycle), speeding up metabolism (Denton, 2009). If the Ca^2+^ concentration is oscillatory, then ATP production in this case will be facilitated during each active phase, leading to ATP/ADP oscillations. A third way for Ca^2+^ to influence metabolism is due to Ca^2+^ flux through the mitochondrial Ca^2+^ uniporter, the main path for Ca^2+^ transport across the mitochondrial inner membrane (De Stefani et al., 2015). This influx depolarizes the mitochondrial membrane potential, reducing ATP synthesis through oxidative phosphorylation (Magnus and Keizer, 1998). If the Ca^2+^ concentration oscillates, then there will be increased flux across the mitochondrial inner membrane during each active phase of the oscillation, resulting in oscillations in ATP synthesis and thus in cytosolic ATP/ADP.

### Symbiosis of the Two Subsystems Provides Multiple Mechanisms for Rhythm Generation

The symbiotic relationship between the metabolic and Ca^2+^ subsystems described here, with mutual feedback from one subsystem to the other, provides for several potential mechanisms underlying electrical bursting and oscillations in Ca^2+^ and metabolism. In one mechanism, oscillations can be generated purely within the metabolic subsystem, and project out to the other subsystems (blue arrow, Figure 2). This intrinsic metabolic oscillator can be mediated by glycolysis, due to the allosteric enzyme phosphofructokinase (PFK), which is positively regulated by its product FBP. This positive autoregulation can lead to oscillations due to the negative feedback that occurs through substrate pool depletion (Smolen, 1995). Indeed, evidence for this comes from work done on skeletal muscle extracts, where the M-type (for muscle) isoform of PFK is dominant (Tornheim and Lowenstein, 1974). This isoform is also dominant in β-cells (Yaney et al., 1995), prompting the proposal that the same rhythmogenic mechanism applies (Tornheim, 1997). We refer to these intrinsic metabolic oscillations as *Active Metabolic Oscillations (AMOs)*. [We previously referred to them as Active Glycolytic Oscillations (Bertram et al., 2018), but have generalized the nomenclature to make clear that the concept applies to oscillations of *all* metabolites, whether they are part of glycolysis or oxidative phosphorylation].

At the other extreme, metabolic oscillations can be generated purely due to the elevations in Ca^2+^ that occur during each active phase, and downstream effects resulting from that elevation (orange arrow, Figure 2), primarily due to ATP hydrolysis by the Ca^2+^ ATPases (Detimary et al., 1998). We refer to these Ca^2+^-dependent metabolic oscillations as *Passive Metabolic Oscillations (PMOs)*, since the metabolic variables in this case are only responding to Ca^2+^.

In either case, the cell’s electrical activity is ultimately determined by ion channels. Several spiking patterns have been reported in the literature: *fast spiking* with a period of about 100 ms; *fast bursting* with superimposed spikes, with a period of tens of seconds (Meissner, 1976; Henquin and Meissner, 1984); and slow bursting with a period of 5 min or more (Henquin et al., 1982; Cook, 1983). Each electrical pattern is associated with a corresponding oscillation in Ca^2+^. For recent work on the interactions of spikes and bursts see Zmazek et al. (2021). In the case of slow bursting, the most likely channel type for turning burst active phases on and off is the K(ATP) channel (Keizer and Magnus, 1989; Detimary et al., 1998; Magnus and Keizer, 1998). If this is correct, then ATP/ADP oscillations drive bursting through the activation/deactivation of K(ATP) channels. A likely mechanism for fast bursting oscillations is cyclic activation of K(Ca) channels.

**FIGURE 3:**
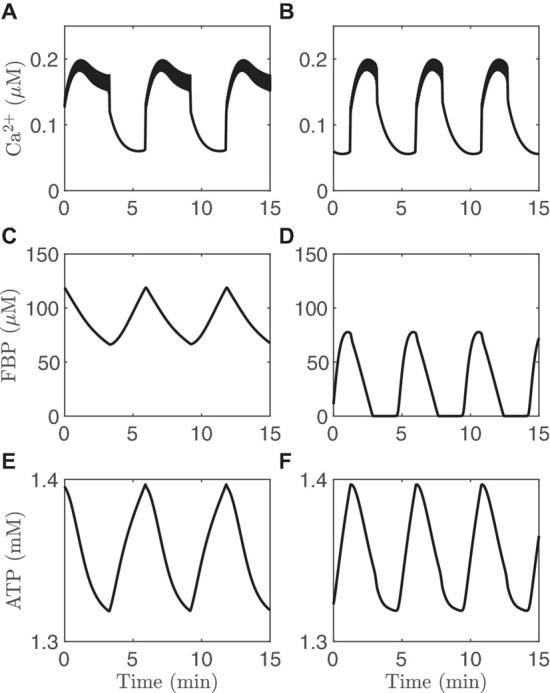
Time courses generated with the IOM. The columns on the left **(A,C,E)** show an example of PMOs, while those on the right **(B,D,F)** show an example of AMOs. For the left column, *v*_PDH_ = 0.4 μM/*ms* while for the right column and *v*_PDH_ = 2 μM/*ms*. In both cases, *g*_K(*Ca*)_ = 150pS and the glucose concentration in 11 mM.

While it can be difficult to ascribe one of these mechanisms or the other to a particular pattern of bursting by period alone, this can be addressed using computer simulations with the IOM, as in Figure 3. The left panels show slow oscillations in which the metabolic oscillations are passive (PMOs), and the right panels show bursting with AMOs. These different scenarios were obtained using different values of the maximum catalytic rate for PDH, *v*_*PDH*_ (Eq. 1). Slow oscillations exist for intermediate range of values of *v*_*PDH*_, as described in Bertram et al. (2018) and explored quantitatively in Marinelli et al. (2018). The Ca^2+^ traces (Figures 3A,B) share the same basic features (a rapid rise at the start of an active phase, a plateau throughout most of the active phase, and a sharp drop initially, followed by a slower drop during the silent phase). The ATP oscillations (Figures 3E,F) also have roughly the same shapes. The only clear difference between the two cases is the shape of the FBP time course. With PMOs it has a sawtooth pattern (Figure 3C) with peaks at the beginning of the active phase and nadirs at the end. With AMOs, FBP is pulsatile (Figure 3D), exhibiting peaks at the beginning of the active phase and then slowly decreasing to almost zero, where it remains during most of the silent phase. While a FRET sensor has been developed in our lab that reports FBP dynamics (PKAR, for Pyruvate Kinase Activity Reporter) (Merrins et al., 2013), our analysis (unpublished observations) of data from a population of islets indicates that it is difficult in practice to experimentally distinguish between the sawtooth and pulsatile FBP patterns.

### Clamping the Ca^2+^ Concentration Can Distinguish Active Metabolic Oscillations and Passive Metabolic Oscillations

**FIGURE 4:**
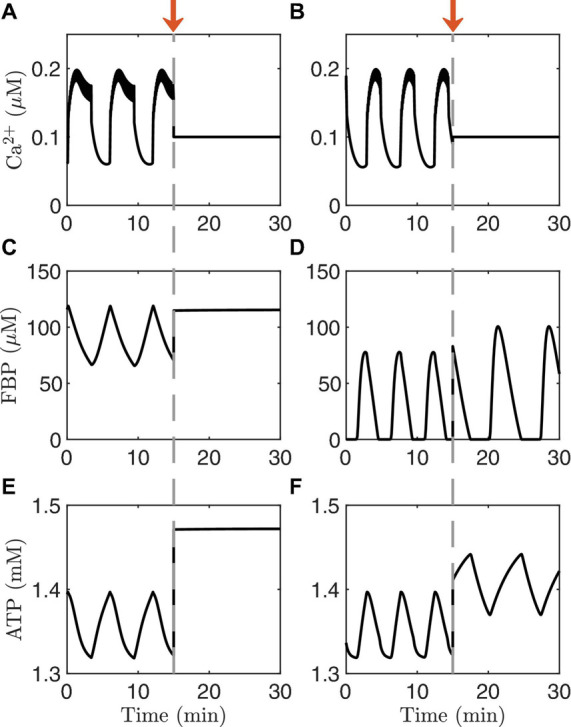
Time courses generated with the IOM. The columns on the left **(A,C,E)** show an example of PMOs, while those on the right **(B,D,F)** show an example of AMOs. At time t = 15 min (arrow), the Ca^2+^ concentration is clamped. In the model with the PMOs (left column) all oscillations stop, while in case of AMOs (right column) the metabolic oscillations persist. The values of *v*_*PDH*_ and *g*_*K(Ca)*_ and glucose are the same as in Figure 3.

Another way to distinguish AMOs and PMOs theoretically is illustrated in Figure 4. As in the previous figure, the traces on the left are from an oscillating system exhibiting PMOs, while those on the right are from a system exhibiting AMOs. At time *t=15* min, the Ca^2+^ concentration is clamped in both cases to a fixed level. This terminates the metabolic oscillations in the case of PMOs (left), while metabolic oscillations persist in the case of AMOs (right). This is a clear qualitative difference between the two oscillation mechanisms that does not require distinguishing between small changes in the shapes of variables. It is a perfect model prediction. But can it be tested experimentally?

**FIGURE 5:**
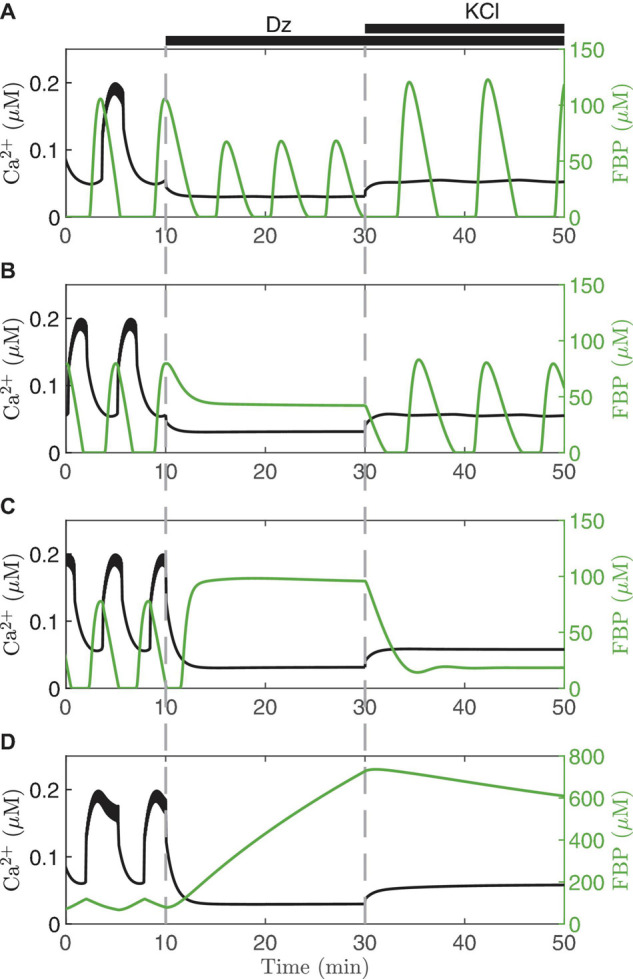
Model simulations in which the membrane potential is clamped with diazoxide (Dz) and elevated KCl. Each panel illustrates the model predictions for *v*_PDH_ = 7 μM/*ms* **(A)**, 3 μM/*ms* **(B)**, 2 μM/*ms* **(C)**, 0.4 μM/*ms* **(D)**. In all cases, *g*_K(*Ca*)_ = 150pS and the glucose concentration is 11 mM.

It is possible to clamp the Ca^2+^ level of a single cell pharmacologically by bath applying diazoxide (Dz), an activator of K(ATP) channels. The level at which Ca^2+^ is clamped, moreover, can be raised by increasing the concentration of KCl in the bath solution, which depolarizes the cells and thereby opens more Ca^2+^ channels. This procedure for clamping islet Ca^2+^ has been used to better understand intracellular signaling in islet β-cells by short-circuiting the dynamics of the Ca^2+^ subsystem (Gembal et al., 1992; Merrins et al., 2010). Model simulations depicting this are shown in Figure 5. Each panel of the figure shows a simulation of what can happen when Ca^2+^ is first clamped with Dz, and then the Ca^2+^ level is increased by raising the KCl concentration in the continued presence of Dz. We illustrate the results in different cases, represented by different values set for the parameter *v*_*PDH*_. The first three panels exhibit AMOs prior to clamping, while the last exhibits PMOs.

When Dz is first applied, the Ca^2+^ oscillations stop in all four cases (Ca^2+^ is clamped to a low value since the cell is hyperpolarized due to K(ATP) opening). However, in the top case (Figure 5A) the metabolic oscillations (shown here by the glycolytic metabolite FBP) persist in Dz. When KCl is applied with Dz, the Ca^2+^ level is increased, and at this higher Ca^2+^ level the metabolic oscillations become slower and larger in amplitude. This is a clear demonstration that, in this case, the metabolic oscillations are AMOs, as in the right panels of Figure 4. They thus do not require oscillatory Ca^2+^ for them to occur, although they do require Ca^2+^ to be at a sufficiently high, but not too high, level (achieved by adding KCl). We also note that the Ca^2+^ level in the presence of Dz does modulate AMO period and amplitude. What is perhaps more surprising is that in the middle two cases (Figures 4B,C), which both also show AMOs, clamping Ca^2+^ with Dz can be seen to terminate the metabolic oscillations. Indeed, a similar finding done experimentally [where NAD(P)H or oxygen consumption was measured as the metabolic variable] led to the conclusion that metabolic oscillations are not intrinsic (AMOs), but must be driven by the Ca^2+^ oscillations (PMOs) (Kennedy et al., 2002; Luciani et al., 2006). As our simulation in Figure 4B shows, however, when Ca^2+^ was subsequently elevated to a higher level, the metabolic oscillations re-emerged. Thus, in this case, metabolic oscillations clearly do not require Ca^2+^ *oscillations*, just a more elevated level of Ca^2+^. Measurements of NAD(P)H were made to test this prediction (Merrins et al., 2010), and it was found that while Dz often terminated the metabolic oscillations, in some of the cases the oscillations re-emerged following the addition of KCl. Thus, the experimental data showed examples that matched both panels A and B of the simulations in Figure 5. A later experimental study also found recovery of the metabolic oscillations with the application of KCl, but in this case measurements were made using PKAR (Merrins et al., 2016), a more direct readout of glycolytic oscillations.

The bottom two panels of Figure 5 show examples where the metabolic oscillations were terminated by simulated application of Dz, and did not recover even following islet depolarization by KCl. This behavior was also observed experimentally (Merrins et al., 2010, 2016). However, it is not known in this case whether the metabolic oscillations present before Ca^2+^ was clamped were AMOs (as in Figure 5C) or PMOs (as in Figure 5D). The latter type would be expected from Figure 4 (left column), as these passive metabolic oscillations must stop when Ca^2+^ does not oscillate. It is harder to understand the former case (Figure 4C) since the metabolic oscillations that existed before Ca^2+^ was clamped were AMOs that do not require Ca^2+^ oscillations. So why were the metabolic oscillations not rescued by elevating Ca^2+^ with KCl? Put simply, it is because the Ca^2+^ level induced by the KCl in this case was not sufficiently large; increasing the Ca^2+^ level further indeed rescues the metabolic oscillations. For more details (see Watts et al., 2014; Marinelli et al., 2018).

**FIGURE 6:**
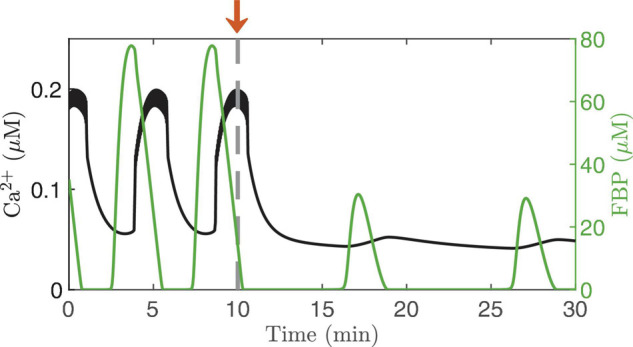
Ca^2+^ oscillations generated by the IOM, with FBP pulses superimposed. The glucose concentration is lowed from 11 to 5 mM at time t = 10 min. The electrical activity stops at the subthreshold glucose concentration, but intrinsic oscillations persist, resulting in small-amplitude Ca^2+^ fluctuation. For this simulation, *v*_PDH_ = 2 μM/*ms* and *g*_K(*Ca*)_ = 150pS.

Islet Ca^2+^ can also be clamped by exposing islets to glucose concentrations below the threshold for electrical activity. This was done in Nunemaker et al. (2006) and small oscillations in the Ca^2+^ concentration could be detected even in the absence of electrical activity. Figure 6 illustrates the model prediction when the concentration of glucose was reduced from a stimulatory level (11 mM) to a substimulatory level near the threshold (5 mM, arrow). At 5 mM, the cell is not bursting, although small fluctuations in Ca^2+^ concentration occur. Where do these fluctuations come from? Examination of the superimposed FBP trace shows that even in subthreshold glucose, intrinsic metabolic oscillations are produced that act on the Ca^2+^ concentration by causing oscillations in ATP/ADP (blue arrow, Figure 2); these produce small voltage fluctuations that result in small Ca^2+^ fluctuations. Note that these subthreshold Ca^2+^ fluctuations are much smaller than the oscillations produced at a stimulatory glucose level, and are too small to drive any plausible Ca^2+^-dependent oscillation mechanism, but they are large enough to be detected experimentally, as shown in Nunemaker et al. (2006).

The subthreshold Ca^2+^ oscillations are likely also too small to drive the oscillations that occur in basal insulin secretion (Satin et al., 2015). They are very slow, which suggests they are the product of intrinsic metabolic oscillations, and it is likely that the metabolic oscillations drive the oscillations in basal secretion. They would do this directly through increasing delivery of insulin granules to the plasma membrane and to the vicinity of the small number of Ca^2+^ channels that open spontaneously even when membrane potential is low. This is supported by observations in super-threshold glucose with Ca^2+^ clamped and glucose artificially pulsed (Ravier et al., 1999; Kjems et al., 2002). The oscillations of secretion under these conditions were only about one-tenth the size of those when Ca^2+^ was free to oscillate, but that would be sufficient to account for oscillations in basal secretion. When Ca^2+^ is free to oscillate, the metabolic oscillations synergize with Ca^2+^ to markedly amplify the secretory pulses, as modeled in Figure 6 of Satin et al. (2015).

Direct confirmation of metabolic oscillations in basal glucose through measurements of a metabolite such as NAD(P)H, FBP, or ATP/ADP remains to be done. This is a particularly tricky experiment since, from an analysis of the model, subthreshold AMOs do not always occur. That is, the result will likely vary from islet to islet, so that many trials might be needed to firmly establish that subthreshold AMOs occur. Indeed, this could be why subthreshold Ca^2+^ oscillations have so rarely been reported in the islet literature. Regardless, subthreshold glucose is an excellent condition in which to bypass the cell’s electrical activity and isolate oscillations in the metabolic subsystem.

### Compound Oscillations Demonstrate the Coexistence of Intrinsic Metabolic Oscillations and Ca^2+^-Driven Oscillations

**FIGURE 7:**
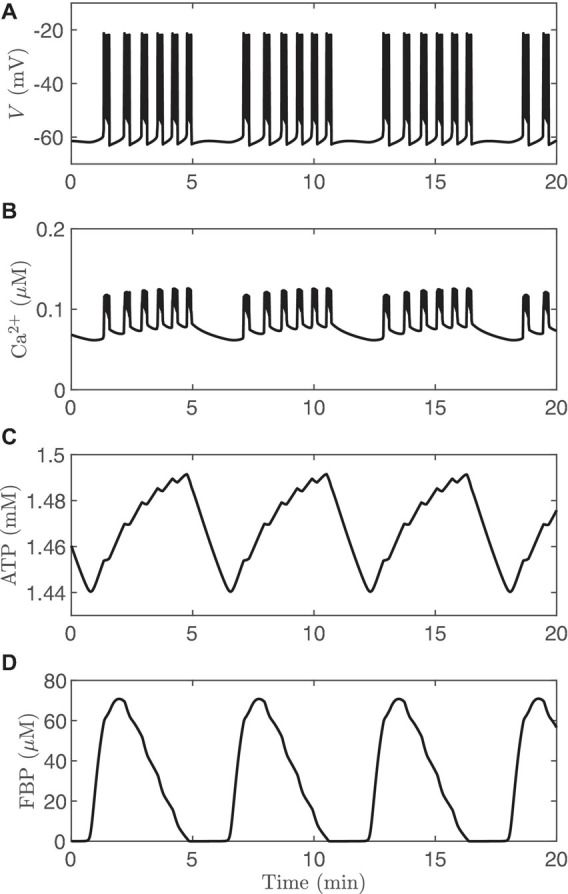
Compound bursting generated by the IOM, and the calcium time courses are similar (Figures 9A,B). **(A)** Membrane potential. **(B)** Cytosolic calcium. **(C)** ATP. **(D)** FBP. For this simulation, *v*_PDH_ = 2 μM/*ms*, *g*_K(*Ca*)_ = 600pS, and the glucose concentration in 11 mM.

The observation that islets exhibit compound electrical or Ca^2+^ oscillations is the strongest evidence to date that β-cells have intrinsic metabolic oscillations. Compound oscillations consist of fast *V* bursts (or Ca^2+^ oscillations) that are grouped into slower episodes. This type of oscillatory pattern has been observed since the earliest reports of islet electrical activity (Henquin et al., 1982; Cook, 1983), and has been confirmed using Ca^2+^ imaging (Valdeolmillos et al., 1989; Bertram et al., 2004) or using simultaneous recordings of islet membrane potential and Ca^2+^ (Beauvois et al., 2006). The period of the burst episodes is about 5 min, corresponding closely to the period of insulin pulsatility. We have hypothesized that the fast oscillations within compound oscillations reflect the feedback of intracellular Ca^2+^ onto K(Ca) channels, while intrinsic slow metabolic oscillations group the fast oscillations into slower episodes (Bertram et al., 2004, 2007b). The mechanism of compound oscillations can be thought of as a combination of the first mathematical model of bursting in β-cells (Chay and Keizer, 1983) [for the fast bursts, and based on K(Ca)] with the first model in which bursting (for the slow burst episodes) relied upon intrinsic metabolic oscillations (Tornheim, 1997). Since both mechanisms are incorporated into the IOM, this model can produce compound oscillations, as shown in Figure 7. Here it can be seen that pulses of FBP (Figure 7D) lead to rises in ATP (Figure 7C) that are responsible for packaging bursts (Figure 7A) and fast Ca^2+^ oscillations (Figure 7B) into episodes via the activation and deactivation of K(ATP) channels. The FBP pulses in this simulation result from AMOs, and the effect of Ca^2+^ on the AMOs is seen only as the “teeth” in the ATP profile. The elevated Ca^2+^ concentration that accompanies each fast burst leads to increased ATP hydrolysis by Ca^2+^ ATPases (orange arrow in Figure 2), resulting in a downward deflection in the ATP trace shown. Such “teeth” have been observed for other metabolic variables and for glucose consumption during compound bursting (Jung et al., 1999; Dahlgren et al., 2005).

**FIGURE 8:**
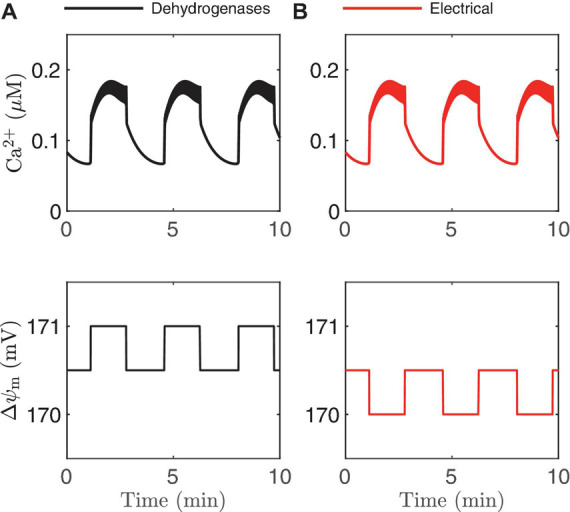
Schematic of experimental findings on the mitochondrial membrane potential △ψ_m_ If the dehydrogenase effects prevail, △ψ_m_ hyperpolarizes during the active phase **(A)**, while △ψ_m_ depolarizes during the active phase if the electrical effect dominates **(B)**.

**FIGURE 9:**
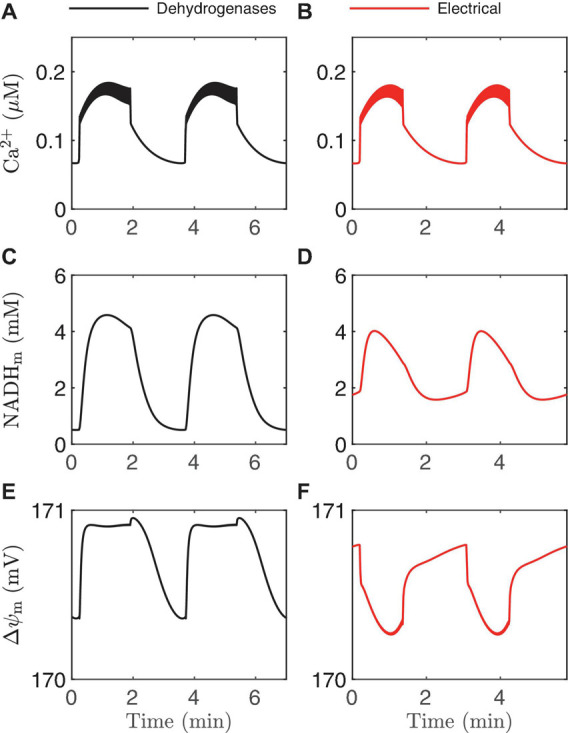
Time courses generated by the IOM with *p*_21_ = 0.013μM/*ms* **(A,C,E)** and 0.03μM/*ms* **(B,D,F)**. In both cases, *v*_PDH_ = 0.4 μM/*ms*, *g*_K(*Ca*)_ = 150pS, and the glucose concentration in 11 mM. In the left panel, the effect of Ca^2+^ on dehydrogenases dominates, while on the right the electrical effect of Ca^2+^ current across the inner membrane dominates.

Although we know of no other reasonable explanation for compound bursting, we cannot rule out other possibilities. Thus, although the existence of compound oscillations is suggestive of AMOs, as both metabolism and Ca^2+^ are oscillating during compound bursting, disentangling the effects of one on the other experimentally is difficult. This highlights why clamping Ca^2+^, either pharmacologically or by using subthreshold glucose remains the best approach for unequivocally distinguishing between AMOs from PMOs experimentally.

### The Model Can Be Used to Resolve Contradictory Behaviors

Models can also be useful for interpreting data from experiments *post hoc*. This is particularly important when the data defy straightforward explanations, or when data from different labs appear to be contradictory. As an example of this, we consider the measurements that have been made of mitochondrial inner membrane potential (△ψ_m_) during bursting. The inner membrane potential is very important as it provides the electrochemical driving force needed for mitochondrial ATP synthesis. In one study, the measurement of △ψ_m_ using the fluorescent dye rhodamine-1,2,3 showed that △ψ_m_ *depolarized* during each burst active phase (Krippeit-Drews et al., 2000), decreasing mitochondrial electrochemical driving force. In a later study, measurements made using the same technique found that △ψ_m_ *hyperpolarized* during each active phase, increasing the electrochemical driving force (Merrins et al., 2016). How can one interpret these apparently contradictory findings? How can they both be right?

In Figure 2 we illustrated two ways that Ca^2+^ can influence ATP production: through the activation of Ca^2+^-dependent dehydrogenases which hyperpolarize △ψ_m_, and via the direct depolarization of △ψ_m_ by the Ca^2+^ current flowing across the mitochondrial inner membrane through the Ca^2+^ uniporter. During a burst active phase, cytosolic Ca^2+^ concentration is elevated, and when some of this Ca^2+^ enters the mitochondria it will thus likely have *both* hyperpolarizing and depolarizing effects on △ψ_m_. Which effect dominates will determine the direction of △ψ_m_ during each burst active phase, as illustrated in Figure 8. If the activation of dehydrogenases by Ca^2+^ dominates, then △ψ_m_ will hyperpolarize during the active phase (left panels, increases in △ψ_m_ indicate hyperpolarization), while if the electrical effect of the Ca^2+^ flux dominates, then △ψ_m_ will depolarize during the active phase (right panels).

This result is intuitive, and modeling is not needed to make the point. What is not clear, however, is what system parameter controls this competition? A key parameter that can change the effect of mitochondrial Ca^2+^ influx during the active phase from △ψ_m_hyperpolarization to depolarization (Bertram et al., 2006) is *p*_*21*_, which appears in the term for Ca^2+^ uniporter flux,


(5) $$J_{\text{uni}}=\left(p_{21}\,△\,\psi_{m}-p_{22}\right)\,{\text{Ca}}^{2+},$$

where Ca^2+^ is the cytosolic Ca^2+^ concentration. In the uniporter flux function the term in parentheses is the effect of △ψ_m_ on Ca^2+^ influx across the mitochondrial inner membrane. The relationship is approximately linear in the relevant range of △ψ_m_, with slope *p*_*21*_ and intercept *p*_*22*_ (Bertram et al., 2007a). For small values of *p*_*21*_, the electrical effect produced is small and the dehydrogenase effect will therefore dominate so that the inner membrane hyperpolarizes during the burst active phase (Figure 9E). Increasing *p*_21_increases the electrical effect, to the point where it will dominate the dehydrogenase effect. As a result, the inner membrane potential now depolarizes during each burst active phase (Figure 9F). In both cases, NADH_*m*_ peaks near the beginning of the active phases (Figures 9C,D), as shown experimentally in Merrins et al. (2016). Thus, measurements of NAD(P)H fluorescence alone cannot predict the effects of Ca^2+^ on △ψ_m_. In summary, islet-to-islet variation in this single parameter, *p*_*21*_, can account for the different findings that have been reported concerning the relationship between burst active phase and △ψ_m_ (Krippeit-Drews et al., 2000; Merrins et al., 2016). Again, this reinforces the utility of modeling in understanding and interpreting published data in the literature.

## Discussion

In this perspectives article, we illustrated that there is a symbiotic relationship between the metabolic and electrical/calcium subsystems of pancreatic β-cells. What couples these systems together in a way that is different from most other cells are the K(ATP) channels, which are found in cardiac myocytes (Noma, 1983), smooth muscle cells (Hibino et al., 2010), a small population of glucose-sensing neurons in the hypothalamus (Burdakov et al., 2005), glucagon-like-peptide-1-secreting L-cells of the small intestine (Reimann and Gribble, 2002), in addition to β-cells (Ashcroft and Rorsman, 2013). While it is widely appreciated that K(ATP) channels are a key player in the transduction of blood glucose level to insulin secretion by β-cells (Ashcroft and Rorsman, 2013), their role in the generation of insulin pulsatility remains a matter of debate. Our goal in this perspectives article was to illustrate the several interacting signaling pathways that exist in β-cells and their influence on electrical bursting oscillations, oscillations in cytosolic Ca^2+^ concentration, and metabolism, the sum of which produces pulsatile insulin secretion.

**FIGURE 10:**
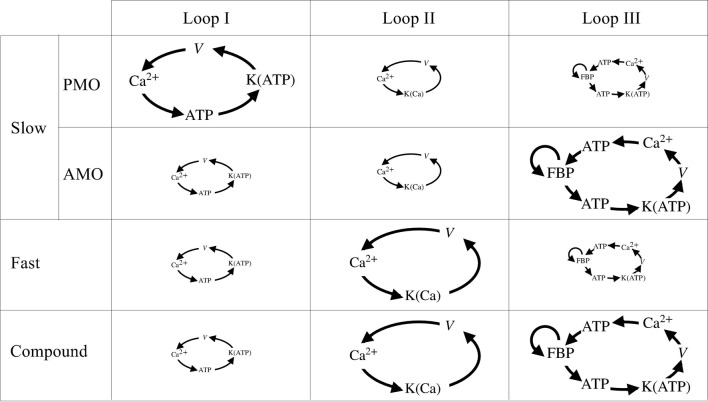
Schematic of the three main loops encapsulating the symbiosis between metabolism and Ca^2+^ activity. The size of each loop is proportional to the importance of its role played in slow, fast, and compound bursting. Note that slow bursting can be driven by two different mechanisms, loop I for PMOs or loop III for AMOs.

Figure 10 summarizes these feedback loops and their contributions to slow, fast, and compound bursting oscillations. The first of these loops (loop I) describes the action of Ca^2+^ on ATP production and consumption through hydrolysis, dehydrogenases, and the mitochondrial Ca^2+^ uniporter. Oscillations in the Ca^2+^ concentration produce oscillations in ATP that are carried forward to the K(ATP) conductance, which in turn, influences the cell membrane potential. The loop is closed by the influence of the membrane potential on voltage-dependent Ca^2+^ channels, which determine the cytosolic Ca^2+^ concentration through Ca^2+^ influx. This feedback loop is responsible for slow bursting oscillations associated with PMOs in the IOM, and is represented as a large loop for the slow oscillations. While the loop is still active during fast and compound oscillations and slow oscillations with AMOs, we propose that it plays a lesser role in either, so we depict it as being small in the diagram.

The second loop (loop II) describes the action of cytosolic Ca^2+^ on K(Ca) ion channels. Activation of these channels has a hyperpolarizing effect on the cell’s membrane potential, which in turn closes *V*-dependent Ca^2+^ channels, reducing the cytosolic Ca^2+^ concentration. We propose that while this loop has only a minor role to play in slow bursting, it is the main driver of fast bursting, including fast bursting that occurs during each episode of compound bursting.

The third feedback loop (loop III) describes the positive feedback effect of FBP on PFK, which produces intrinsic glycolytic oscillations (AMOs). These glycolytic oscillations result in oscillations in ATP, which affect the membrane potential again through K(ATP) channels. The electrical bursting results in Ca^2+^ oscillations, which affect the cell’s ATP level, which in turn affects PFK since ATP is an allosteric inhibitor of PFK (Passonneau and Lowry, 1962). Loop III has the potential to produce slow bursting with AMOs in our model, and we believe it is responsible for the slow wave underlying compound bursting as well as slow sub-threshold Ca^2+^ oscillations. It is unlikely to be active during fast bursting and slow bursting with PMOs, however.

It is our belief that the vast majority of observed phenomena regarding oscillations in β-cell activity in mouse islets, where most of the research on islet oscillations has been performed, can be understood from the standpoint of these three canonical feedback loops. Because all three are incorporated into the IOM, the model is a useful tool for determining the conditions that must hold to explain various phenomena. This article focused on a few specific behaviors that can be interpreted with the use of the model; some others have been the focus of prior reports (see Bertram et al., 2018 for a recent review). The success of the IOM stems from the fact that it incorporates feedback pathways that can account for the symbiotic relationships observed among the metabolic, electrical, and Ca^2+^ subsystems of β-cells.

## Author Contributions

IM performed mathematical simulations and analysis. PF provided conceptual advice on the model. AS, LS, and RB provided resources and supervision. All authors contributed to writing the manuscript.

## Conflict of Interest

The authors declare that the research was conducted in the absence of any commercial or financial relationships that could be construed as a potential conflict of interest.

## Publisher’s Note

All claims expressed in this article are solely those of the authors and do not necessarily represent those of their affiliated organizations, or those of the publisher, the editors and the reviewers. Any product that may be evaluated in this article, or claim that may be made by its manufacturer, is not guaranteed or endorsed by the publisher.
